# β-Adrenergic Receptor Inhibitor and Oncolytic Herpesvirus Combination Therapy Shows Enhanced Antitumoral and Antiangiogenic Effects on Colorectal Cancer

**DOI:** 10.3389/fphar.2021.735278

**Published:** 2021-10-15

**Authors:** Jiali Hu, Cuiyu Chen, Ruitao Lu, Yu Zhang, Yang Wang, Qian Hu, Wanting Li, Shiyu Wang, Ouyang Jing, Hanying Yi, Wei Zhang, Ling Chen, Weihua Huang, Jia Luo, Howard L. McLeod, Ran Xu, Yijing He

**Affiliations:** ^1^ Department of Clinical Pharmacology, Xiangya Hospital, Central South University, Changsha, China; ^2^ Hunan Key Laboratory of Pharmacogenetics, Institute of Clinical Pharmacology, Central South University, Changsha, China; ^3^ Zhuhai People’s Hospital (Zhuhai Hospital Affiliated with Jinan University), Zhuhai, China; ^4^ School of Basic Medical Sciences, Guangzhou Medical University, Guangzhou, China; ^5^ Department of Gastrointestinal Surgery, Xiangya Hospital, Central South University, Changsha, China; ^6^ Department of Hepatobiliary and Intestinal Surgery, Hunan Cancer Hospital, Changsha, China; ^7^ Geriatric Oncology Consortium, Tampa, FL, United States; ^8^ Department of Urology, The Second Xiangya Hospital of Central South University, Changsha, China

**Keywords:** propranolol, oncolytic virus, colorectal cancer, apoptosis, antiangiogenesis

## Abstract

Oncolytic viruses (OVs) are considered a promising therapeutic alternative for cancer. However, despite the development of novel OVs with improved efficacy and tumor selectivity, their limited efficacy as monotherapeutic agents remains a significant challenge. This study extended our previously observed combination effects of propranolol, a nonselective *β*-blocker, and the T1012G oncolytic virus into colorectal cancer models. A cell viability assay showed that cotreatment could induce synergistic killing effects on human and murine colorectal cell lines. Moreover, cotreatment caused sustained tumor regression compared with T1012G monotherapy or propranolol monotherapy in human HCT116 and murine MC38 tumor models. The propranolol activity was not via a direct effect on viral replication *in vitro* or *in vivo*. Western blotting showed that cotreatment significantly enhanced the expression of cleaved caspase-3 in HCT116 and MC38 cells compared with the propranolol or T1012G alone. In addition, propranolol or T1012G treatment induced a 35.06% ± 0.53% or 35.49% ± 2.68% reduction in VEGF secretion in HUVECs (*p* < 0.01/*p* < 0.01). Cotreatment further inhibited VEGF secretion compared with the monotherapies (compared with propranolol treatment: 75.06% ± 1.50% decrease, compared with T1012G treatment: 74.91% ± 0.68%; *p*＜0.001, *p* < 0.001). Consistent with the *in vitro* results, *in vivo* data showed that cotreatment could reduce Ki67 and enhance cleaved caspase 3 and CD31 expression in human HCT116 and murine MC38 xenografts. In summary, *β*-blockers could improve the therapeutic potential of OVs by enhancing oncolytic virus-mediated killing of colorectal cancer cells and colorectal tumors.

## Introduction

With the approval of Talimogene laherparepvec (T-VEC) by the US FDA in 2015 (Kohlhapp and Kaufman, 2016), the use of oncolytic viruses (OVs) to treat tumors has become a promising area of cancer therapy development. OVs can selectively replicate in tumor cells and induce apoptosis without harming adjacent normal tissues (Kaufman et al., 2015). A large number of preclinical studies have confirmed that OVs have activity against a variety of solid tumors, through direct oncolytic killing effects and enhance antitumor immune responses, including against colorectal cancer (Liu et al., 2010; Amagai et al., 2016; Huang et al., 2016; Yang et al., 2016). Despite the development of novel OVs with improved efficacy and tumor selectivity, the limited efficacy of OVs as monotherapeutic agents remains a significant challenge (Kirn et al., 1998; Andtbacka et al., 2015).

Propranolol, a nonselective β-blocker, can exert antitumor effects by inducing apoptosis, including effects on colorectal cancer (Masur et al., 2001; Liao et al., 2010; Zhou et al., 2014; Liao et al., 2020). OVs produce a number of cytopathic effects, including apoptosis (Gaddy and Lyles, 2007; Cary et al., 2011; Garant et al., 2016; Xiao et al., 2018; Lal and Rajala, 2019). OVs and propranolol therapies are likely to be synergistic when combined, since they trigger cell death through distinct pathways. Together, they may promote an enhanced killing effect on tumor cells.

Propranolol has also been known to block tumor angiogenesis in infantile hemangioma (Drolet et al., 2013; Léauté-Labrèze et al., 2017). In addition to its antitumor effect, propranolol has been shown to exert antiangiogenic activities (Liu et al., 2015). At present, there is expanding evidence that wildtype HSV (Strain F) and its derivatives G47Δ and d120 exert antiangiogenic effects on different tumors (Mahller et al., 2007; Liu et al., 2008; Hardcastle et al., 2010; Wu et al., 2019), and T1012G was obtained by single knocking γ34.5 on the basis of wild type F strain. Therefore, we speculate that T1012G combined with propranolol may exert synergistic antitumor and antiangiogenic effects on colorectal cancer. Using T1012G as a model OV, we tested our hypothesis in colorectal cancer cell lines and engrafted mouse models.

## Materials and Methods

### Cell Lines, Virus and Reagents

HCT116, Widr, MC38, CT26WT, HUVEC and vero cells were obtained from the American Type Culture Collection (Manassas, VA). In addition to CT26WT cell line (cultured in RPMI 1640 medium (Gibco, Life Technologies, China) supplemented with 10% FBS), the other cell lines were cultured in DMEM medium (Gibco, Life Technologies, China) supplemented with 10% FBS (HCT116 and MC38) or 5% newborn calf serum (Vero cell) (Gibco, Life Technologies, Australia) at 37°C and 5% CO 2 in tissue culture incubator. The virus T1012G was obtained by single knocking γ34.5 on the basis of wild type F strain (Yan et al., 2019). Phase II clinical trials evaluating NV1020, an oncolytic herpesvirus (oHSV), have been completed in the US and showed safety and effectiveness in patients with colon cancer or liver cancer (Geevarghese et al., 2010). T1012G, studied in this project, includes a deletion of the inserted HSV-2 glycoprotein based on NV1020, which could reduce the pathogenicity of the virus (Yan et al., 2019). In addition, using T1012G as the backbone, which carries human IL-12 and anti-PD-1 antibody genes, a new OV called T3011 was developed and is now being studied in three phase I clinical trials in United States, Australia and Shanghai, China simultaneously (NCT04370587).

### Cell Viability Assay

The cells were seeded in a 96-well plate at a seeding density of 2,500–3,000 cells/well. After 24 h, the cells were treated with propranolol or virus T1012G. After 48 h of treatment, the liquid in the well plate was aspirated and CCK8 activity detector was added into to the wells. Then the plate should be avoided the light and placed in a 37°C incubator, 30–60 min later, the plate was placed in a microplate reader and tested at a wavelength of 450 nm.

### Hoechst Staining

Cells treated with propranolol, T1012G, propranolol plus T1012G were fixed, washed twice with PBS and stained with Hoechst 33258 staining solution according to the manufacturer’s instructions (Beyotime, Jiangsu, China). Stained nuclei were observed under a fluorescence microscope (Celigo® Image Cytometer, United States).

### Apoptosis Analysis Using Propidium Iodide and Annexin V Staining

For apoptosis analysis using PI and Annexin V, HCT116 cells were seeded in 6-well plates (3 × 10^5^ cells per well) and co-treated with T1012G (MOI 0.01, 0.05, and 0.1) and propranolol (80 μM). At the end of the 2 days incubation period, the cells were collected by trypsinization, transferred into 15 ml tubes, and washed with ice-cold PBS. After washing, the cells were resuspended in binding buffer containing 1μl/ml PI and 1 μl/ml Annexin V APC-conjugated (Beyotime, Jianfsu, China). Apoptosis analysis, which was based upon the cell surface exposure of phosphatidyl serine that binds to Annexin V, was performed using the Beckman coulter FC500 flow cytometer (Beckman Coulter Inc.).

### Western Blot Analysis

Western blot analysis was performed on cell extracts of HCT116 cell lines treated with 80 μM propranolol plus different dose of virus (0.01, 0.05, 0.1 MO) for 48 h. Immunoblots were performed from whole cell lysate prepared using RIPA Buffer supplemented with dithiothreitol (DTT), and fresh protease and phosphatase inhibitors (Sigma). Cell lysates were quantified for protein content using a bicinchoninic acid (BCA) protein assay kit (Beyotime, Jiangsu, China). Protein samples were resolved on NuPAGE 12% Bis-Tris gels with MOPS buffer or 3–8% Tris acetate gels with Tris acetate buffer (Life Technologies) and then transferred to 0.45-mm nitrocellulose membrane (Bio-Rad). After saturation in Tris-buffered saline supplemented with 5% BSA, the membranes were incubated with antibodies (diluted at 1:2,000) overnight at 4°C. Antibody specific for the following proteins were purchased from Abcam: cleaved-caspase3 (rabbit, ab2302). The antibody specific for GAPDH (rabbit, KM9002) was purchased from Sungene Biotech. The antibody specific for β-actin (mouse, 66009-1-Ig) was purchased from proteintech. Quantification of the bands was done with ImageJ

### *In Vitro* and *Vivo* Viral Replication

*In vitro*, propranolol at low toxicity concentrations was used and the virus was stored in milk 24 or 48 h after treatment with different sequential drugs and viruses in tumor cells. After repeated freezing and thawing three times, the virus was added to the pre-paved vero cells. After 3 days of infection, the number of plaques was calculated, and the virus concentration under different treatments was obtained. In the *in vivo* HCT116 animal model, tumor tissues were collected 15 days after the last intratumoral injection of the virus, and the virus titer in the tumor tissues under different treatments was detected.

### CD31/Ki67/Cleaved Caspase-3 Immunohistochemistry and Quantification

Mice (*n* = 6/group) were treated as described above and at day 17 post-implantation tumors were harvested, frozen and cut into tissues sections. Tissue sections were stained with CD31 antibody (abcam, ab281583)/Ki67 antibody (abcam, ab15580)/cleaved caspase -3 (abcam, ab184787) followed by secondary anti-rat IgG conjugated to HRP (GE Healthcare, Piscataway, NJ). CD31+/Ki67+/cleaved caspase-3+ staining was revealed with 3,3′-diaminobenzidine (DAB) histochemistry (Vector Laboratories, Burlingame, CA). Sections were counterstained with hematoxylin (Sigma, St. Louis, MO). Tumor microvessel density was quantified for all treatment groups. At least 6–10 representative 40X fields per view were captured as epifluorescent digital images using a Spot digital camera (Spot Diagnostic instruments, Sterling, MI). To calculate microvessel density, area occupied by CD31-positive microvessels and total tissue area, per section were quantified using ImageJ software (NIH, Bethesda, MD). Microvessel density was then calculated as a percentage of CD31 stained per tumor section.

### Animal Studies

HCT116 cells was engrafted into BALB/C nude mice. About 70–80% of mice developed solid tumors in 5–7 days. Mice were divided into six groups including: blank control (PBS); propranolol (2 mg/kg for a week; 6 mg/kg from the 9th to 11th day); T1012G (5 × 10^5^ pfu/mouse was injected intratumorally on the first day), and three combined treatment groups with different administration orders. The simultaneous treatment is the same as the single respective treatments. In the pretreatment with propranolol combined group, propranolol was administrated in the same way as the single drug treatment and then injected intratumorally on the 8th day. In the pretreatment with virus combined group, the virus was administered in the same way as the virus only group and propranolol began to be administered on the third day. The administration cycle and dose were consistent with the drug-only group.

Four to five weeks of C57 were inoculated subcutaneously with 5 × 10^5^ mc38 cell suspension, and about 1 week later, the tumor volume reached 80–120 mm^3^. Then the mice were divided into six groups: the control group, two single drug groups and three combined treatment groups. The drug and or virus were administered for 7 days in the single drug groups. Among them, propranolol (2 mg/kg) was administered continuously for 1 week, and virus T1012G (1 × 10^7^ pfu/mouse) was administered once every 2 days for a total of three administrations. In addition, the two combination groups treated in sequence were treated with another drug or virus after the end of the administration of the virus or propranolol. Tumor volume and mouse body weight were measured during the period. The study protocol was approved by the Ethics Committee of Xiangya Hospital, Central South University (No. 2020sydw0167) and all experiments were performed in accordance with approved guidelines of Xiangya Hospital, Central South University.

### Determination of Apoptosis Induction by Annexin V/PI Staining

HCT116 or MC38 cells were exposed to propranolol and/or T1012G (80 μM + 0.05 MOI/60 μM + 0.1 MOI) for 48 h and then stained with Annexin V and propidium iodide (PI) to determine the proportion of apoptotic cells by flow cytometry (BeckmanCoulter, America).

### VEGF Detection

HUVECs were treated with propranolol and/or T1012G (100 μM + 0.1 MOI) for 24 h. Culture medium was collected and analyzed for the VEGF concentration by ELISA (R&D, SVE00).

### Statistical Analysis

Data were presented as mean ± SEM. Significant differences were evaluated using one-way ANOVA or unpaired t-test. Differences were considered significant if the *p* value was less than 0.05. All statistical analyses were performed using GraphPad Prism software (GraphPad Software, Inc., version 8.0).

## Results

### Cotreatment With T1012G and Propranolol Exerted a Synergistic Killing Effect on Human Colorectal Cancer Cells and Endothelial Cells

**FIGURE 1:**
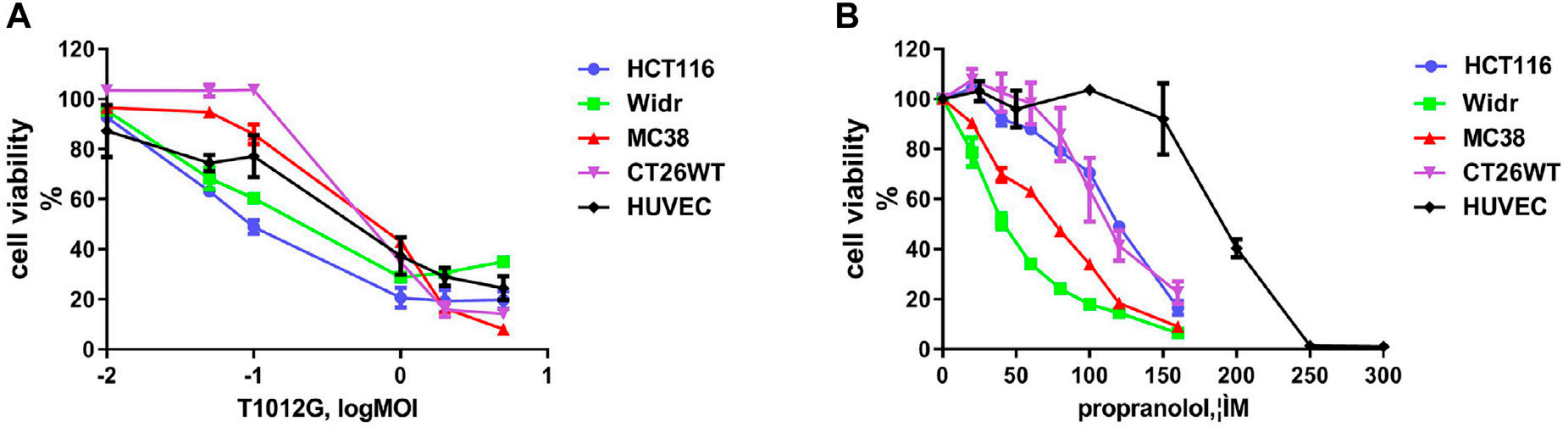
Effects of T1012G and propranolol as separate agents on cell proliferation in colorectal cancer cell lines and human umbilical vein endothelial cells (HUVECs). **(A,B)** A CCK-8 assay was used to measure cell viability at 48 h after increasing the doses of T1012G (0.01, 0.05, 0.1, 1, 2, and 5 MOI; 0.01, 0.05, 0.1, 1, 2, and 5 MOI) and propranolol (20, 40, 60, 80, 100, 120, and 160 μM; 25, 50, 100, 150, 200, 250, and 300 μM) used to treat colorectal cancer cells and endothelial cells, respectively. Results are presented as the mean ± SEM.

**FIGURE 2:**
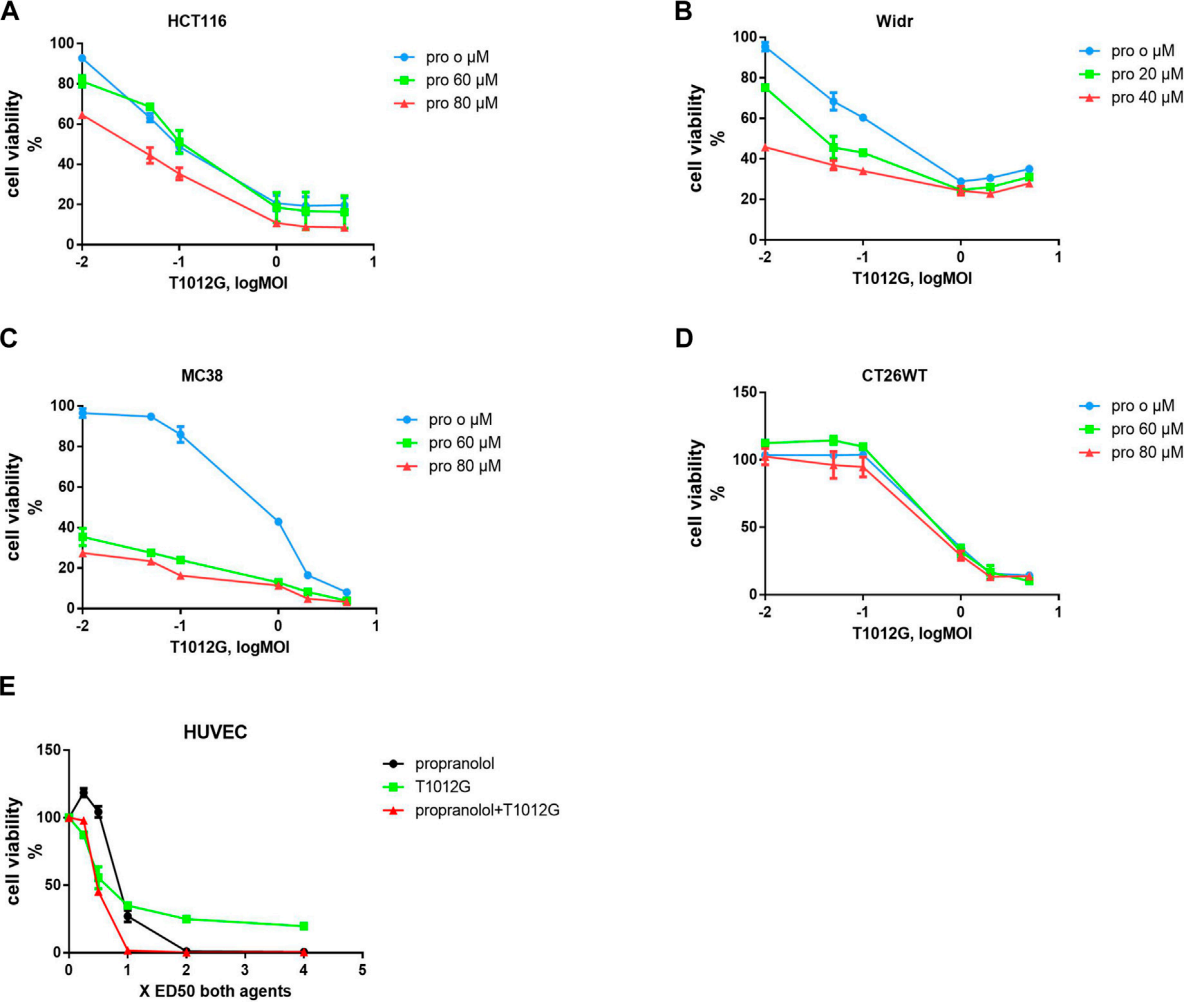
Combined treatment effects on the proliferation of colorectal cell lines and endothelial cells. **(A–D)** A CCK-8 assay was used to calculate the survival rate after 48 h of combined treatment with T1012G (0.01, 0.05, 0.1, 1, 2, or 5 MOI) and propranolol (60 or 80 µM in HCT116, MC38 and CT26WT cells; 20 or 40 µM in Widr cells). **(E)** HUVECs were exposed to propranolol and T1012G at 0.25, 0.5, 1, 2 and 4× the calculated IC_50_. Results are presented as the mean ± SEM. Pro, propranolol.

The IC_50_ values of T1012G were determined to be 0.14, 0.35, 0.64, 0.74, and 0.48 MOI for the HCT116, Widr, MC38, and CT26WT cell lines and human umbilical vein endothelial cells (HUVECs), respectively, and the IC_50_ values of propranolol were 116, 42, 69, 115, and 192 μM in these cell lines (Figure 1). Combined therapy with these two agents exhibited enhanced inhibition of cell viability in two human colorectal cancer cell lines, one murine colorectal cancer cell line and HUVECs (Figures 2A–C, E). The synergistic effect was measured by the combination index (CI) using the Chou-Talalay algorithm (Chou et al., 1991). The lowest CI values (0.671, 0.504, and 0.455) were observed in the cotreatment groups at 80 μM + 1 MOI, 20 μM + 1 MOI and 80 μM + 2 MOI in the HCT116, Widr and MC38 colorectal cancer cell lines, respectively (Table 1). In addition, with the concomitant administration of T1012G and propranolol, there was evidence of synergism with CI values at ED50, ED75, ED90 and ED95 of 0.88, 0.66, 0.59, and 0.58 in HUVECs, respectively (Table 2). Cotreatment with the two agents exerted antagonistic effects on CT26WT murine colorectal cancer cells (Figure 2D; Table 1).

**TABLE 1:** Combination index (CI) values for propranolol and T1012G combination treatment of colorectal cancer cell lines.

HCT116		Widr	
Combination dose	CI^a^	Combination dose	CI^a^
60 μM + 0.01 MOI	1.319 ± 0.070	20 μM + 0.01 MOI	0.991 ± 0.264
60 μM + 0.05 MOI	1.679 ± 0.114	20 μM + 0.05 MOI	0.511 ± 0.162
60 μM + 0.10 MOI	1.246 ± 0.187	20 μM + 0.10 MOI	0.516 ± 0.107
60 μM + 1.00 MOI	1.184 ± 0.531	20 μM + 1.00 MOI	0.504 ± 0.107
60 μM + 2.00 MOI	1.790 ± 1.175	20 μM + 2.00 MOI	0.836 ± 0.047
60 μM + 5.00 MOI	3.490 ± 2.391	20 μM + 5.00 MOI	2.588 ± 0.393
80 μM + 0.01 MOI	0.929 ± 0.049	40 μM + 0.01 MOI	0.849 ± 0.194
80 μM + 0.05 MOI	0.864 ± 0.130	40 μM + 0.05 MOI	0.702 ± 0.070
80 μM + 0.10 MOI	0.805 ± 0.046	40 μM + 0.10 MOI	0.695 ± 0.107
80 μM + 1.00 MOI	0.671 ± 0.148	40 μM + 1.00 MOI	0.729 ± 0.004
80 μM + 2.00 MOI	0.694 ± 0.162	40 μM + 2.00 MOI	0.897 ± 0.139
80 μM + 5.00 MOI	1.019 ± 0.444	40 μM + 5.00 MOI	2.249 ± 0.082
**MC38**		**CT26WT**	
**Combination dose**	**CI1**	**Combination dose**	**CI1**
60 μM + 0.01 MOI	0.737 ± 0.103	60 μM + 0.01 MOI	13.92 ± 9.694
60 μM + 0.05 MOI	0.637 ± 0.036	60 μM + 0.05 MOI	15.75 ± 13.97
60 μM + 0.10 MOI	0.607 ± 0.016	60 μM + 0.10 MOI	19.68 ± 17.65
60 μM + 1.00 MOI	0.627 ± 0.003	60 μM + 1.00 MOI	1.084 ± 0.002
60 μM + 2.00 MOI	0.583 ± 0.031	60 μM + 2.00 MOI	1.137 ± 0.115
60 μM + 5.00 MOI	0.505 ± 0.092	60 μM + 5.00 MOI	1.869 ± 0.282
80 μM + 0.01 MOI	0.809 ± 0.050	80 μM + 0.01 MOI	4.492 ± 1.327
80 μM + 0.05 MOI	0.749 ± 0.048	80 μM + 0.05 MOI	3.145 ± 1.218
80 μM + 0.10 MOI	0.611 ± 0.038	80 μM + 0.10 MOI	3.816 ± 1.665
80 μM + 1.00 MOI	0.685 ± 0.021	80 μM + 1.00 MOI	1.236 ± 0.048
80 μM + 2.00 MOI	0.455 ± 0.111	80 μM + 2.00 MOI	1.204 ± 0.090
80 μM + 5.00 MOI	0.496 ± 0.174	80 μM + 5.00 MOI	2.360 ± 0.168

a Combination index ( CI < 1: synergism; CI = 1: additive; CI > 1: antagonism). The CI values of the combination models were measured by the Chou-Talalay method, where the CI value quantitatively defines synergism (CI < 1), additive effects (CI = 1) and antagonism (CI > 1). Data are presented as the mean ± SEM.

**TABLE 2:** Combination index (CI) values for propranolol and T1012G combination treatment of human umbilical vein endothelial cells.

HUVEC				
	ED50	ED75	ED90	ED95
CI	0.879 ± 0.121	0.661 ± 0.128	0.585 ± 0.148	0.583 ± 0.173

Propranolol treatment did not have significant effect on viral replication in colorectal cancer cell lines *in vitro*.. T1012G and propranolol were combined at 0.25, 0.5, 1, 2, and 4 times the ED50 of each agent using a constant ratio design. Combination indices were generated using CompuSyn software. Data are presented as the mean ± SEM.

### Propranolol Treatment Did Not Have Significant Effect on Viral Replication in Colorectal Cancer Cell Lines *In Vitro*

**FIGURE 3:**
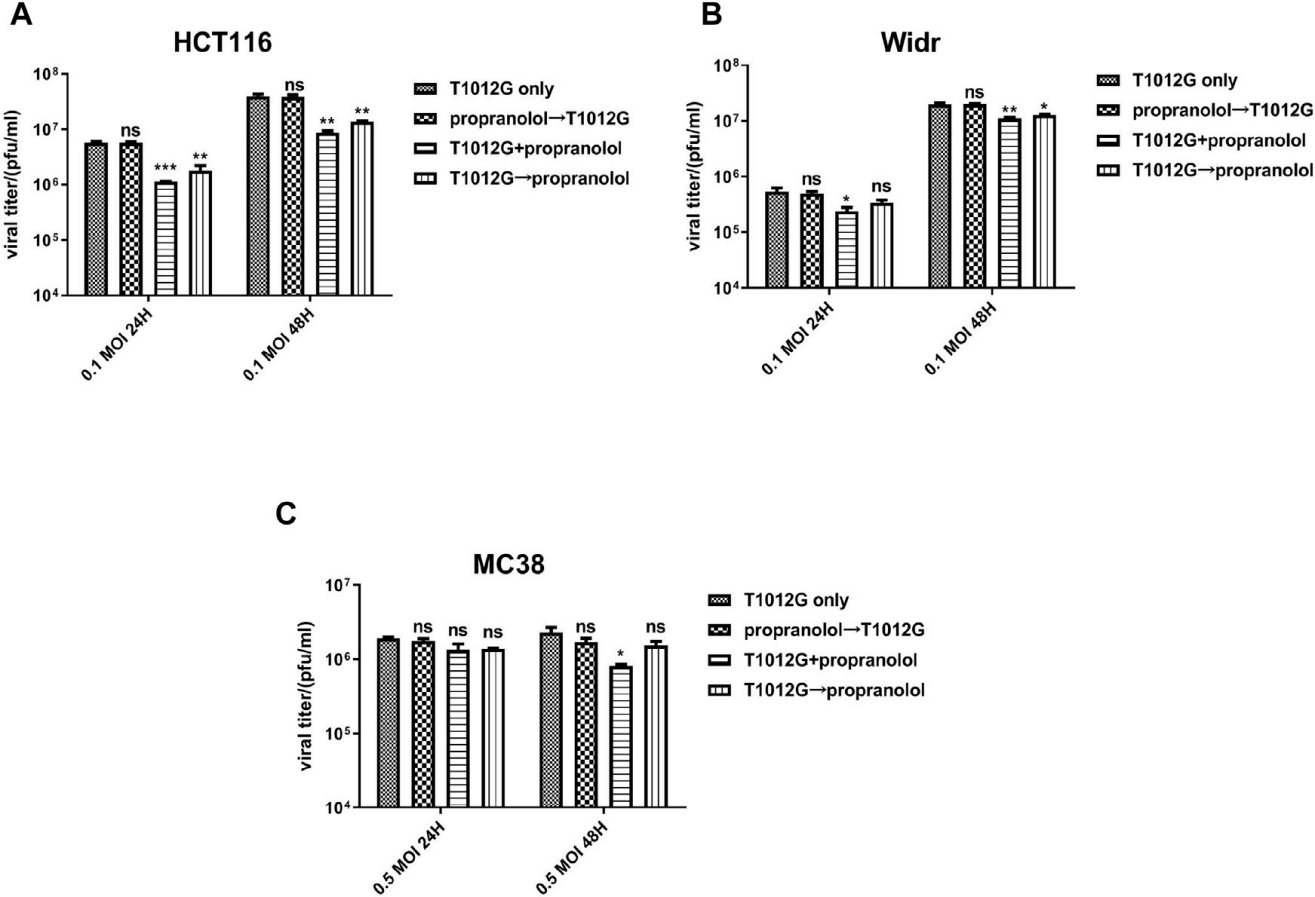
Combined treatment effects on viral replication in colorectal cell lines *in vitro*. **(A–C)** A viral replication assay was applied to measure the propagation of T1012G (0.1, 0.1, or 0.5 MOI) in HCT116, Widr and MC38 cells cotreated, pretreated or post treated with propranolol (60, 20, or 20 μmol/l), respectively. The titer of T1012 was measured 1 and 2 days after infection. Data are presented as the mean ± SEM. *p < 0.05, **p < 0.01, ***p < 0.001 vs. T1012 only (Dunnett’s multiple comparison test). Combined T1012G and propranolol treatment enhanced tumor growth delay in human and murine colorectal models.

Cotreatment with propranolol (60 or 20 μmol/l) and T1012G (0.1 MOI) and pretreatment with virus significantly attenuated viral replication in the human HCT116 and Widr colorectal cancer cell lines (Figures 3A,B). However, propranolol pretreatment did not affect virus replication (Figures 3A,B, *p* > 0.05). These results indicated that the synergistic killing effects on the two human cancer cell lines induced by simultaneous cotreatment were not caused by affecting viral replication. In addition, cotreatment could reduce viral replication in only MC38 cells (Figure 3C, *p* < 0.05 (48 h)), while sequential treatment exerted no significant effect on viral propagation (Figure 3C, *p* > 0.05).

### Combined T1012g and Propranolol Treatment Enhanced Tumor Growth Delay in Human and Murine Colorectal Models

**FIGURE 4:**
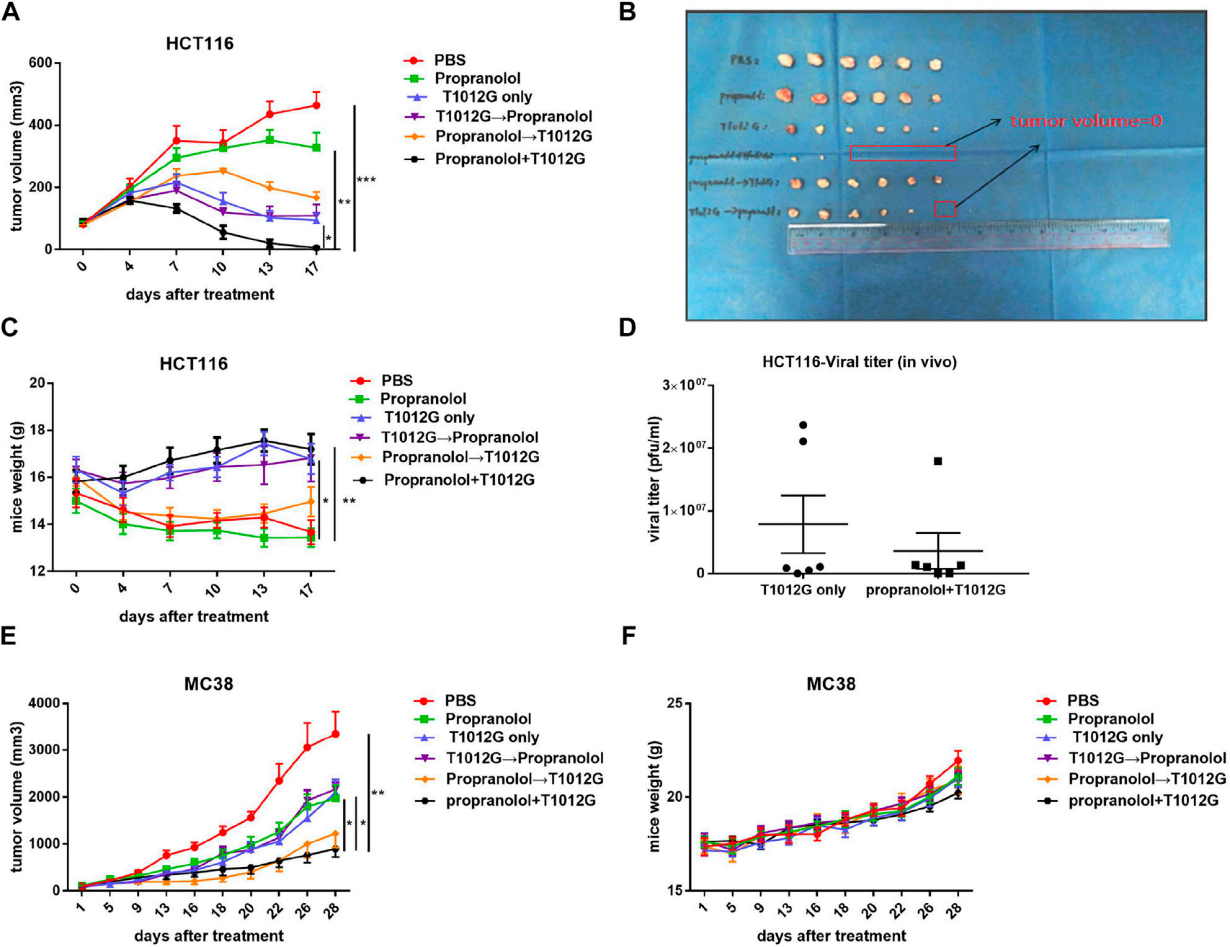
Combined treatment inhibited tumor development. **(A,B)** Tumor growth curves show the average volume of 6 tumors in each group for the human HCT116 colorectal tumor model. *p < 0.05, **p < 0.01, ***p < 0.001 vs. propranolol+T1012G (Dunnett’s T3 multiple comparison test). **(C)** HCT116 model mouse body weights were measured on day 0 and every 3 days thereafter. *p < 0.05, **p < 0.01 vs. PBS (Dunnett’s T3 multiple comparison test). **(D)** Titers of progeny virus in tumors were determined by standard plaque assays using Vero cells. *p* > 0.05 (unpaired t-test). **(E)** Tumor growth curves show the average volume of 6-8 tumors in each group for the murine MC38 colorectal tumor model. **(F)** MC38 model mouse body weights were measured at the beginning of the test and every 3 days thereafter. Results are presented as the mean ± SEM. Significant differences were evaluated using one-way ANOVA or an unpaired t-test. Combination treatments inhibited proliferation, induced apoptosis and enhanced antiangiogenic activity in human and murine colorectal xenografts.

The synergistic effect of propranolol and T1012G was assessed in HCT116 tumors engrafted in BALB/C nude mice. Only the tumor volumes of the coadministration group were smaller than those of the monotherapy groups, and more than half of the tumors regressed (5.60 ± 3.74 vs. 327.20 ± 49.36 mm^3^, *p* < 0.01; 5.60 ± 3.74 vs. 94.69 ± 22.24 mm^3^, *p* < 0.05; Figures 4A,B). In addition, mouse weight gradually decreased during the course of the experiment in the blank control group, while that in the cotreatment group increased (17.20 ± 0.65 vs. 13.67 ± 0.51 g, *p* < 0.01; Figure 4C). These results indicated that the mouse survival might be improved after simultaneous administration. There was no difference in virus titer in the tumor between the cotreatment group and the T1012G-only group in the animal model (4.3 × 10^6^ ± 3.4 × 10^6^ vs. 7.9 × 10^6^ ± 4.6 × 10^6^ pfu/ml, *p* > 0.05; Figure 4D).

In a murine MC38 colorectal tumor model, among the treatment groups, only cotreatment inhibited the increase in tumor volume compared with PBS, propranolol and T1012G monotherapies (893.8 ± 171.8 vs. 3349 ± 479.9 mm^3^, *p* < 0.01; 893.8 ± 171.8 vs. 1972 ± 283.6 mm^3^, p < 0.05; 893.8 ± 171.8 vs. 2095 ± 278.4 mm^3^, *p* < 0.05; Figure 4E). Additionally, none of the mice in the treatment groups lost body weight (Figure 4F), which suggested the low toxicity of the combination treatment.

### Combination Treatments Inhibited Proliferation, Induced Apoptosis and Enhanced Antiangiogenic Activity in Human and Murine Colorectal Xenografts

**FIGURE 5:**
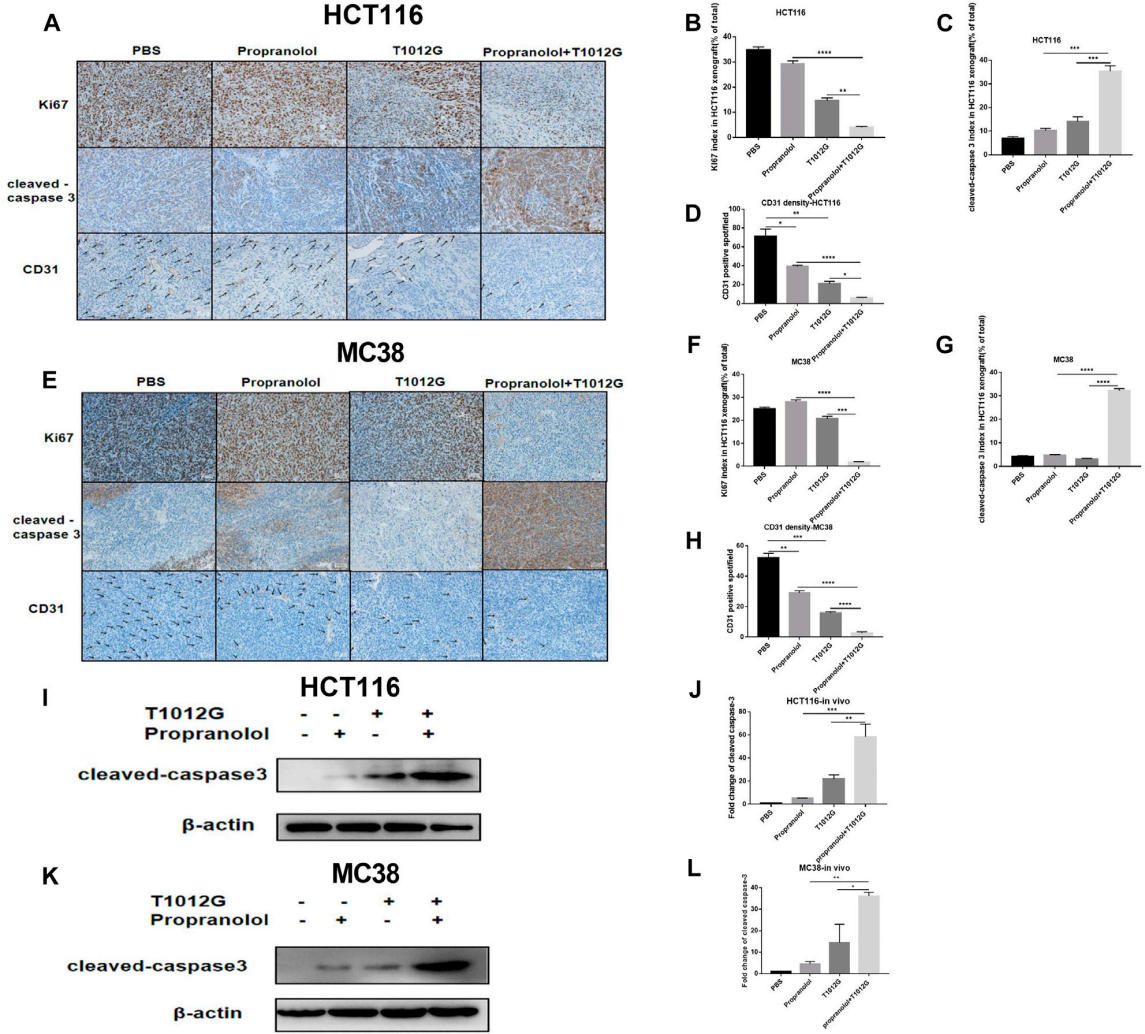
The effect of combined treatment on tumor histology *in vivo*. **(A–E)** Ki67, cleaved caspase-3 and CD31 were assessed by immunohistochemistry. **(I–K)** Western blot analysis of cleaved caspase-3 in PBS-, propranolol-, T1012G-, and propranolol + T1012G-treated tumor tissues. **(B–D, F–H)** Quantification of Ki67/cleaved caspase-3/CD31 staining in HCT116 xenograft mice. **(J–L)** Quantification of the data in **(I, K)**. Results are presented as the mean ± SEM, and significant differences were evaluated using one-way ANOVA. *p < 0.05, **p < 0.01, ***p < 0.01 and ****p < 0.0001 (Tukey test for multiple comparisons). **p < 0.01 and ***p < 0.01 (Games-Howell’s multiple comparisons test). Enhanced apoptosis and VEGF inhibition with combination therapy.

We assessed the proliferation level indicated by the cell marker Ki67 in tumor sections. The Ki67 index was significantly decreased in the coadministration group in human HCT116 and murine MC38 xenografts compared with the monotherapy treatment groups (*p* > 0.0001, *p* < 0.01; *p* > 0.0001, *p* < 0.001; Figures 5A, B, E, F). The expression of cleaved caspase-3 was used to detect the effect of the combination treatment on apoptosis. The expression of cleaved caspase-3 was strongly induced after cotreatment with T1012G and propranolol compared with either monotherapy in HCT116 and MC38 xenografts (*p* < 0.001, *p* < 0.001; *p* < 0.0001, *p* < 0.0001; Figures 5A, C, E, G). We also found that the expression of cleaved caspase-3 in cotreatment group tumor tissues was significantly upregulated (HCT116: 11.36-fold compared with propranolol treatment, *p* < 0.001; HCT116: 2.68-fold compared with T1012G treatment, *p* < 0.01; MC38: 7.88-fold compared with propranolol treatment, *p* < 0.01; MC38: 2.51-fold compared with T1012G treatment, *p* < 0.05; Figures 5I–L). The antiangiogenic effect of the combined therapy was tested using anti-CD31 antibody staining, which marks endothelial cells, of HCT116 and MC38 tumor tissue sections. Propranolol and T1012G monotherapies exerted antiangiogenic effects (*p* < 0.05, *p* < 0.01; *p* < 0.01, *p* < 0.001; Figures 5A, D, E, H). Combination treatment further reduced tumor vascularity compared to the single agents (*p* < 0.0001, *p* < 0.05; *p* < 0.0001, *p* < 0.0001; Figures 5A, D, E, H).

### Enhanced Apoptosis and VEGF Inhibition With Combination Therapy

**FIGURE 6:**
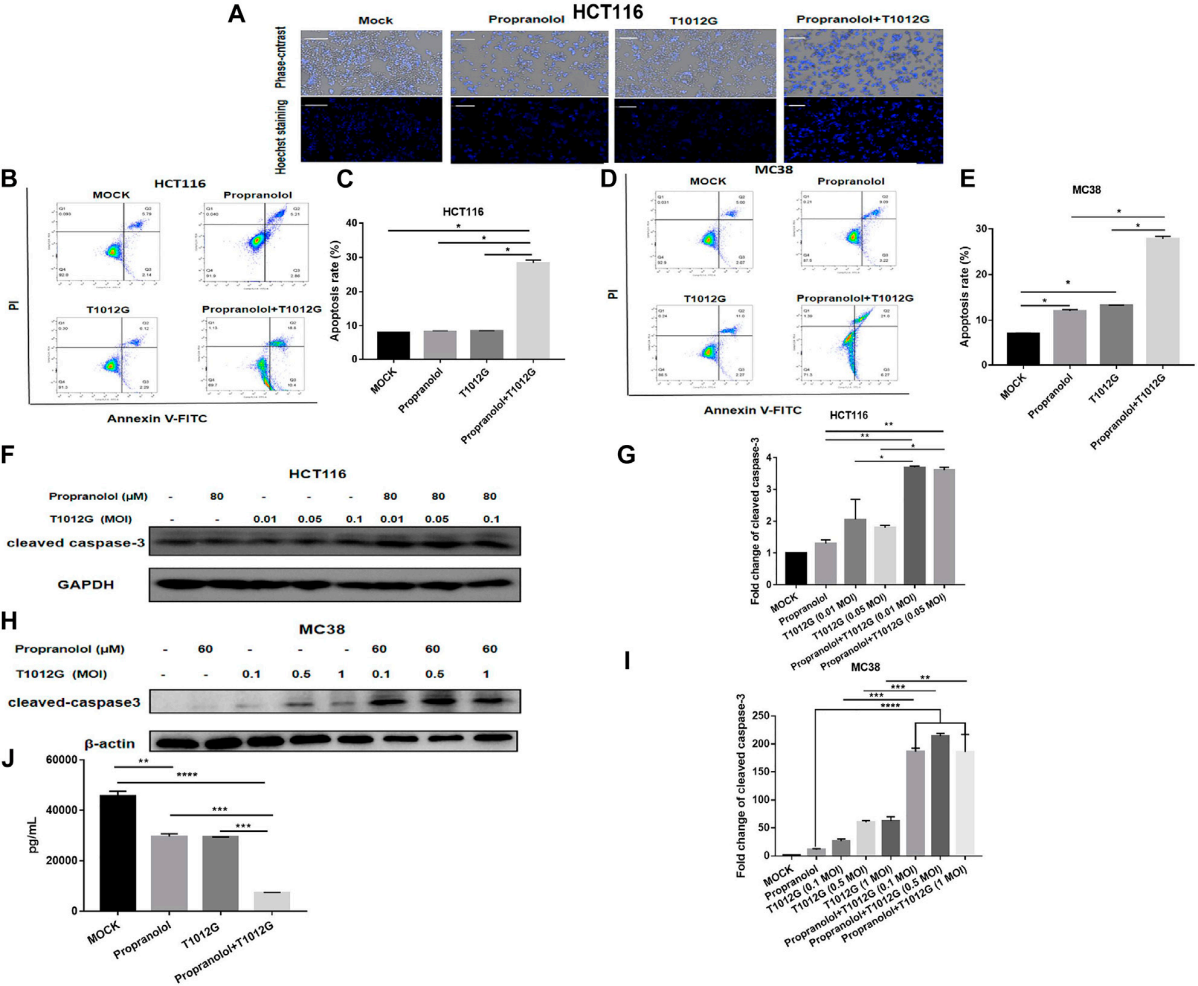
Combined treatment induced apoptosis in human colorectal cancer cell lines. **(A)** Hoechst staining was performed on the HCT116 cell line after combination treatment (80 μM + 0.05 MOI) or single-drug treatment. **(B–D)** HCT116 or MC38 cells were exposed to propranolol and/or T1012G (80 μM + 0.05 MOI/60 μM + 0.1 MOI) for 48 h and then stained with Annexin V and propidium iodide (PI) to determine the proportion of apoptotic cells by flow cytometry. **(F–H)** The expression of cleaved caspase-3 following exposure to 80 μM/60 μM propranolol with different doses of virus (0.01 MOI, 0.05 MOI, or 0.1 MOI or 0.1 MOI, 0.5 MOI, or 1 MOI) for 48 h in the HCT116 and MC38 cell lines. **(J)** HUVECs were treated with propranolol and/or T1012G (100 μM + 0.1 MOI) for 24 h. Culture medium was collected and analyzed for the VEGF concentration by ELISA. **(C, E, G, I)** Quantification of apoptotic cells and cleaved caspase-3 expression. Results are presented as the mean ± SEM, and significant differences were evaluated using one-way ANOVA. *p < 0.05, **p < 0.01, ***p < 0.01 and ****p < 0.0001 (Tukey test for multiple comparisons).

According to Hoechst staining results, compared with the single-drug treatments, combination therapy induced chromatin concentration, which indicated that cotreatment could induce apoptosis in the HCT116 cell line *in vitro* (Figure 6A). The total number of apoptotic cells was significantly upregulated 3.43-fold/3.36-fold compared with the propranolol/T1012G treatments in the HCT116 cell line (*p* < 0.05, *p* < 0.05; Figures 6B,C). Caspase-3, an initiator caspase in the intrinsic apoptotic pathway, was activated by the combination treatment. Cotreatment significantly increased the expression of the activated form of caspase 3 in HCT116 cells (0.01 MOI/0.05 MOI; compared with propranolol treatment: 2.83-fold/2.76-fold, compared with T1012G treatment: 1.79-fold/2.00-fold; *p* < 0.01/*p* < 0.01, *p* < 0.05/*p* < 0.05; Figures 6F,G). Similar to the results in HCT116 cells, cotreatment also induced an increased number of apoptotic cells and enhanced the expression of cleaved caspase 3 in another colorectal cell line, MC38 (0.01 MOI/0.05 MOI/1 MOI; compared with propranolol treatment: 16.63-fold/19.07-fold/16.58-fold, compared with T1012G treatment: 7.12-fold/3.55-fold/2.97-fold; *p* < 0.0001/*p* < 0.0001/*p* < 0.0001, *p* < 0.001/*p* < 0.001/*p* < 0.01; Figures 6H,I). These data strongly suggested that combinatorial treatment could enhance the apoptosis of colorectal cancer cells by activating the intrinsic apoptotic pathway, which was consistent with the *in vivo* results.

Figure 6J shows that propranolol or T1012G treatment could induce a 35.06% ± 0.53% or 35.49% ± 2.68% reduction in VEGF secretion in HUVECs (*p* < 0.01/*p* < 0.01). Cotreatment further inhibited VEGF secretion compared with the single agents (compared with propranolol treatment: 75.06% ± 1.50% decrease, compared with T1012G treatment: 74.91% ± 0.68%; *p*＜0.001, *p*＜0.001; Figure 6J).

## Discussion

This study elucidated that T1012G and propranolol combination therapy enhanced antitumor efficacy and antiangiogenic effects. We also identified enhanced apoptosis through elevated levels of cleaved caspase 3 as the main mechanism of antitumor activity.

Oncolytic virotherapy has become a potential therapeutic approach in colorectal cancer (Ahn and Bekaii-Saab, 2017; Wu et al., 2019). However, the overall efficiency of OVs is still low, and improving efficiency is a major challenge in the clinic (Kirn et al., 1998; Andtbacka et al., 2015). The current solution focuses on combining an OV with agents that could enhance viral replication. Mohammed G and his colleagues found that pretreatment with ruxolitinib reduced ISG expression and thus enhanced viral replication, making tumors susceptible to oHSV infection (Ghonime and Cassady, 2018). Similar results were also seen in our previous work showing that pretreatment with propranolol could enhance the replication of T1012G and thus improve antitumor efficacy in a gastric cancer model. In this study, cotreatment with propranolol and T1012G resulted in a synergistic killing effect on human and murine colorectal cancer cells and sustained tumor regression in an HCT116 human colorectal cancer model. However, the combination therapy did not possess a positive effect on virus replication *in vitro* or *in vivo*.

OVs can specifically replicate and spread in cancer cells *in situ*, exhibiting oncolytic activity through direct cytopathic effects (apoptosis, etc.) while sparing normal cells (Aubert et al., 1999). Chen-Jei Tai et al. showed that cotreatment induced improved antitumor efficacy through enhancing the apoptosis of breast cancer cells compared to either an oncolytic measles virus or camptothecin alone (Tai et al., 2019). A similar result was reported by Xiao, who found that NU7441 (DNA-PK inhibitor) promotes the DNA damage response induced by M1 virus, leading to increased tumor cell apoptosis. Similar to these findings, our study results demonstrated that cotreatment enhanced apoptosis by increasing the expression of cleaved caspase 3 in HCT116 human and MC38 murine colorectal tumor models, which was consistent with the *in vitro* results. In addition, much evidence has shown that many F strain OVs exert killing effects on endothelial cells (HUVECs, etc.) and inhibit tumor angiogenesis (Liu et al., 2008). T1012G, the strain studied in this project, is also an F strain virus. In this study, we found that T1012G treatment also inhibited VEGF secretion *in vitro* and tumor angiogenesis *in vivo*. Furthermore, cotreatment enhanced antiangiogenic effects, but further studies are needed to unveil the mechanisms.

These are the first data to demonstrate that propranolol treatment can enhance virotherapy in colorectal cancer by enhancing apoptosis and that cotreatment with propranolol and T1012G can significantly inhibit tumor angiogenesis in a colorectal tumor model. The effect of propranolol in combination with an OV on other cancers needs further exploration. In addition, clinical trials are needed to identify the effect of combined treatment with propranolol and a virus in colorectal cancer patients as an adjuvant regimen.

In summary, combination with propranolol may improve the therapeutic potential of OVs by enhancing oncolytic virus-mediated killing of colorectal cancer cells and antiangiogenic effects on tumors.

## Data Availability

The original contributions presented in the study are included in the article/supplementary material, further inquiries can be directed to the corresponding authors.
